# Rupture Pathway of Phosphatidylcholine Liposomes on Silicon Dioxide

**DOI:** 10.3390/ijms10041683

**Published:** 2009-04-17

**Authors:** Erik Reimhult, Bengt Kasemo, Fredrik Höök

**Affiliations:** 1 Department of Applied Physics, Chalmers University of Technology, SE-412 96 Gothenburg, Sweden; E-Mails: kasemo@chalmers.se (B.K.); fredrik.hook@chalmers.se (F.H.); 2 Laboratory for Surface Science and Technology, Department of Materials, Swiss Federal Institute of Technology Zurich (ETH Zurich), CH-8093 Zurich, Switzerland

**Keywords:** Lipid Vesicle, Supported Bilayer, Quartz Crystal Microbalance with Dissipation Monitoring (QCM-D), Adsorption, Asymmetric Labeling

## Abstract

We have investigated the pathway by which unilamellar POPC liposomes upon adsorption undergo rupture and form a supported lipid bilayer (SLB) on a SiO_2_ surface. Biotinylated lipids were selectively incorporated in the outer monolayer of POPC liposomes to create liposomes with asymmetric lipid compositions in the outer and inner leaflets. The specific binding of neutravidin and anti-biotin to SLBs formed by liposome fusion, prior to and after equilibrated flip-flop between the upper and lower monolayers in the SLB, were then investigated. It was concluded that the lipids in the outer monolayer of the vesicle predominantly end up on the SLB side facing the SiO_2_ substrate, as demonstrated by having maximum 30–40% of lipids in the liposome outer monolayer orienting towards the bulk after forming the SLB.

## 1. Introduction

Supported lipid bilayers (SLBs) are planar, two-dimensional, extended bilayers, commonly prepared by a method pioneered by McConnell *et al.* [1]. In this method suspended unilamellar liposomes (lipid vesicles) are exposed to a suitable surface, inducing rupture and fusion of the adsorbed liposomes to a continuous SLB. Along with the recent development of various surface analytical tools, the SLB has emerged as a central platform both as model system for functionality studies of cell-membrane-residing biological entities and as surface modification in biosensor applications. The reason for the latter is the combination of their excellent biological inertness, reducing non-specific binding of most proteins and cells to a minimum [2–4], and the possibility to incorporate a biological function, for example membrane residing or interacting proteins or peptides [2,5–9]. Such functional entities, especially transmembrane proteins, are preferably incorporated in the liposomes prior to surface exposure. Under appropriate conditions, this renders spontaneous formation of SLBs carrying the very same functionality [6,10,11]. This approach requires that the desired orientation of the functional site can be controlled, e.g., by preparing a unidirectional orientation in the proteoliposmes, which is transferred into a unidirectional incorporation also in the SLB. However, the results from this method described in the literature indicate different outcomes in terms of transfer of protein directionality from proteoliposome to SLB.

With this work, we aim to elucidate how the membrane of pure liposomes unfolds during rupture and SLB spreading by investigating how the lipids in the two monolayers of the liposome membrane are distributed between the surface proximal and distal monolayers of the SLB. An answer to this question provides a central key to the understanding of how a unidirectional orientation of proteins in vesicles will translate to an SLB.

SLB formation from liposomes occurs in three successive main phases which can be more or less pronounced (direct single-liposome rupture can occur if the attractive surface interaction is strong enough)[12,13]: (a) initially adsorbed liposomes populate the surface; (b) at a critical vesicle coverage, vesicles start to rupture forming a mixed surface of vesicles, bilayer islands and bare surface; and (c) further growth of the SLB is primarily driven by SLB-edge induced (self-promoting) vesicle rupture. Finally, a continuous SLB is formed covering the entire surface. While no solid theoretical framework exists which predicts the details of this process, Seifert *et al.* have developed a thermodynamic framework for understanding single liposome rupture subject to a surface induced deformation based on the Helfrich membrane model [14]. Although the work by Seifert *et al.* gives a thermodynamic explanation for surface-induced rupture of individual vesicles, the detailed kinetics and dynamics are not elucidated. For example, how rupture is initiated is still speculation, and the role played by neighboring liposomes or how the rupture propagates and the membrane unfolds are still unclear. It is important to note that kinetically complex systems are known to often not follow a minimum energy pathway to an equilibrium state and thermodynamic arguments thus are likely to lead to wrong predictions about the actual process. Regardless of these shortcomings this theory, while not having molecular detail implies that at least 50% of the membrane is expected to orient with the outer monolayers of the vesicles facing the substrate, since the unfolding of the membrane is expected to occur with a large part of the outer membrane already adsorbed on the substrate and the rupture unfolding from a point in the membrane of high curvature in the non-adsorbed part of the membrane. By recent phenomenological Monte Carlo simulation models the kinetics of the rupture and unfolding process have been elucidated for models of POPC vesicles going through the above mentioned phases and also for vesicles with a mixture of charged lipids [15–17]. These models are however based on purely phenomenological interaction potentials and do not address the detailed molecular distribution between the lipid leaflets, for which more experimental input would be needed.

By forming SLBs from vesicles containing transmembrane proteins, a few previous studies have experimentally addressed the question of membrane orientation after vesicle rupture and reached contradicting results. In brief, the conclusion from these studies is that proteins in vesicles, which were exposing the active site to the extra-cellular (bulk) liquid, also exposed this site predominantly (>90%) towards the bulk liquid after SLB formation [6,10], which is unexpected given the theoretical considerations discussed above. However, later studies have reported a ~70% decrease in functionality of incorporated transmembrane proteins after SLB formation, indicating a profound loss in directionality [11]. There have also been direct studies of rupture of protein-free vesicles, indicating that the vesicle membrane unfolds in a way that would make proteins, which are oriented with the active site outwards in the vesicle, to redistribute with equal orientation towards the bulk and the substrate in the SLB [18,19]. In one of these studies Jass *et al.* imaged decomposition of single cholesterol-rich liposomes with atomic force microscopy on Si-wafers, and two scenarios were observed: (a) the upper bilayer of adsorbed, deformed vesicles slides down next to the already adsorbed lower bilayer, as if the vesicle was split in half (most common scenario) or (b) the upper bilayer rolls down as if it is still joined to the bottom bilayer on one side [18].

**Figure 1.**
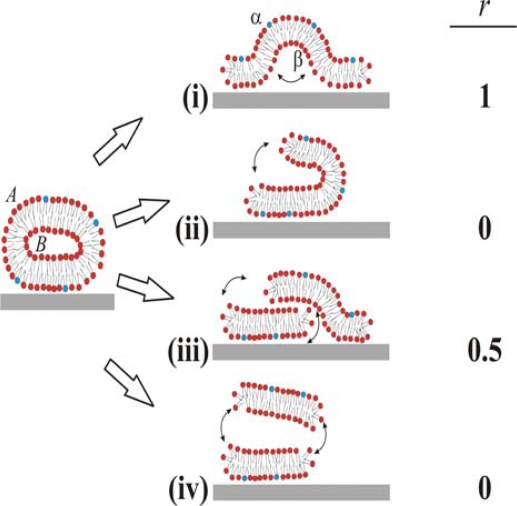
Four possible scenarios for vesicle rupture, unfolding and supported lipid bilayer formation are represented schematically in a simplified 2D side view: (i) rupture forces the membrane outwards, exposing the outer monolayer *A* of the liposome to the bulk, corresponding to monolayer *α* in the SLB; (ii) rupture opens the membrane on one side and unfolding results in exposure of the liposome monolayer *B* to the bulk; (iii) rupture divides the membrane into two fragments which adsorb next to each other, resulting in a ~50:50 ratio of the lipids in *A* between the *α* and *β* monolayer lipids; (iv) displays the same mechanism as in (iii) but the upper membrane fragment desorbs, resulting in an effective complete orientation of the inner leaflet, *B*, of the vesicle towards the bulk, *α*, in the SLB. The right column displays the idealized fraction, *r*, of outer-monolayer lipids, *A*, in the vesicle membrane ending up in the bulk-exposed upper leaflet, *α*, in the adsorbed SLB after completed formation.

Based on these ambiguous experimental and theoretical findings four different pathways for the unfolding of the vesicle membrane on the surface can be identified, as schematically simplified in two dimensions in Figure 1. In pathway (i), the rupture of the membrane must take place where the membrane is in contact with the surface. This part then unrolls outwards, collapsing the vesicle with the outer monolayer predominantly facing the bulk medium (cf. [6,10]). In pathway (ii), rupture starts at the point of highest curvature, and grows to unzip the membrane along the edge. The part of the membrane that is not in contact with the surface is then able to successively roll onto the substrate, making the outer monolayer predominantly face the substrate on the opposite edge from the “unzipping” [18]. Pathways (iii) and (iv) are variations of (ii), where unzipping along the strained edge splits the vesicle into two membrane fragments. In scenario (iii), the top fragment experiences a weak attractive van der Waals interaction from the surface and will, through guided random motion, slide down and adsorb on the surface next to the bottom bilayer fragment in original contact [18]. This scenario would produce roughly 50:50 distribution of the outer monolayer in the vesicle between the upper and lower monolayers in the SLB. In pathway (iv), the attractive surface potential is too weak, allowing for desorption of the upper liposome fragment to the bulk after unzipping. Scenario (iv) will ideally yield the same distribution of lipids as scenario (ii), i.e. the outer monolayer will predominantly face the substrate. These are ideal scenarios and it should directly be pointed out that for a free lipid bilayer patch diffusion of lipids around the edge will equilibrate the lipid distribution between the two monolayers if the membrane is not rapidly expanded. Also, in the proposed scenario for SLB formation (see a-c above), initial rupture of adsorbed vesicles (b) may very well represent a rupture mechanism which is different from the SLB-edge induced vesicle rupture (c), as will be further described in the Discussion.

We denote lipids that belong to the outer and inner monolayers of the liposome as *A* and *B*, respectively, and those belonging to the surface distal and proximal monolayers of the SLB as *α* and *β*, respectively. The different scenarios depicted in Figure 1 differ with respect to the fraction, *r* (shown in the right column in Figure 1), of lipids in monolayer *A* in the liposome that end up in monolayer *α* in the SLB after rupture. We experimentally address the unfolding pathway by selectively incorporating biotin-modified lipids into monolayer *A* of liposomes in solution, and quantifying the fraction of these that ends up in the upper monolayer, *α*, of the SLB. The quantification is performed by measuring streptavidin and anti-biotin binding after SLB formation by quartz crystal microbalance with dissipation monitoring (QCM-D) [20].

## 2. Results and Discussion

SLB formation was monitored by quartz crystal microbalance with dissipation monitoring (QCMD), demonstrating complete bilayer formation from 1.5 mg/mL liposome concentration within <60 s and with asymptotic resonant frequency change (Δ*f*) and dissipation change (Δ*D*) values (Δ*f* = −26 Hz and Δ*D* ≈ 1e-7) indicating only a small population (< 2%) of adsorbed liposomes (not shown) [21]. Subsequent addition of neutravidin (20 μg/mL) or anti-biotin (6.6 μg/mL) was then used to evaluate (titrate) the concentration of biotin-lipids in the upper monolayer facing the bulk, *α*. The biotin-binding molecules were added either (i) immediately (minutes) after SLB formation to determine the initial concentration of biotin-lipids in the substrate distal monolayer *α* of the SLB, *C_α,_*_initial_, or (ii) when trans-bilayer lipid exchange, *i.e.* lipid flip-flop, between monolayers *α* and *β* in the SLB had reached equilibrium with respect to biotin-lipid concentration (hours), *C*_*α*,equilibrium_. The latter state corresponds to an equal biotin-lipid distribution in the upper and bottom monolayers, which equals half the total biotin-lipid concentration: *C*_total_/2 = *C*_*α*,equilibrium_. Thus, since *C*_*α,*initial_, reflects the initial biotin-lipid concentration in the upper monolayer, *α*, immediately after rupture, the fraction, *r*, of lipids in the liposome outer monolayer *A* that ends up in *α* after SLB formation can be determined from:

(1) $$r=\frac{C_{\alpha ,\text{initial}}}{C_{\text{total}}}=\frac{C_{\alpha ,\text{initial}}}{2C_{\alpha ,\text{equlibrium}}}$$

**Figure 2.**
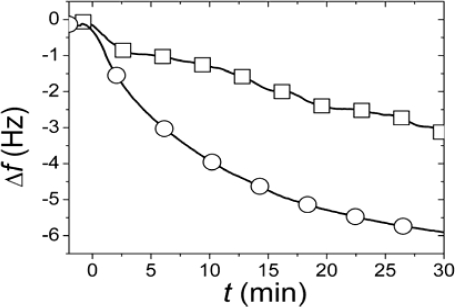
Example adsorption kinetics for the change in resonant frequency Δ*f* (−Δ*f* ∝ Δ*m*) obtained by QCM-D for the adsorption of neutravidin to the SLB formed from liposomes with biotin-lipids selectively incorporated in the outer leaflet measured immediately (<4 min) after the SLB was formed (Δ*f*_initial_, squares) and at 17 h after completed SLB (Δ*f*_equilibrium_, circles).

A change in *C* from immediately after the SLB formation event to when the equilibrium situation has been reached will thus reveal whether and how the asymmetry of the original lipid distribution in the liposome has been transferred to an asymmetry in the SLB. In the first approach used to determine *r*, SLBs were simultaneously formed in two identical measurement cells using the same batch of liposomes and conditions. After completed SLB formation, the vesicle solutions were rapidly exchanged for pure buffer in both measurement cells. While one of the SLBs was then exposed to neutravidin immediately (<4 minutes) after completed SLB formation and rinsing, the other was incubated for >12 h after rinsing before addition of an identical neutravidin suspension. Typical results for the relation in the magnitudes and rates of the changes in *f* upon addition of the neutravidin after equilibrated flip-flop (Δ*f*_equlibrium_) and immediately after SLB formation (Δ*f*_initial_) are shown in Figure 2 [22]. The Δ*f*_initial_ and Δ*f*_equlibrium_ values, proportional to adsorbed neutravidin mass, were then used as *C_α_*_,initial_ and *C*_equilibrium_ respectively in Equation (1), that is

(2) $$r_{NA}=\frac{\Delta f_{\text{initial}}}{2\Delta f_{\text{equilibrium}}}$$

It should be pointed out that the absolute values of the changes in *f* and *D* were relatively sensitive to the preparative steps (the amount of biotin-PE incorporated in the liposomes), but *r*_NA_ = 0.29±0.055 was similar for six independent measurements (error calculated as the standard deviation for the independent measurements), where *r*_NA_ was evaluated at a time of typically 30 min, when the *r*_NA_ was only very slowly changing. These results clearly show that at least 70% of the biotin-lipids in the outer monolayer *A* in the vesicles end up in the monolayer *β* facing the substrate after SLB formation. A comparison of *r*_NA_ with the ratios in Figure 1 yields that this result seems to exclude scenario (i) and requires scenario (ii) to be at least one of the dominating rupture pathways. However, further experiments were performed to verify this hypothesis.

The experiment using neutravidin was done on two different surfaces prepared in parallel, which means that the conclusion relies on individual pair-wise comparison of two different samples, albeit prepared under identical conditions and from the same stocks. In principle, a more precise quantification would be obtained by titration of a biotin-binding molecule to the same sample at several time-points during the lipid redistribution process. In order to test this hypothesis, IgG mouse anti-biotin was used instead of neutravidin because of its reduced single-site dissociation constant of ~10^7^ M^−1^, which is less than the square root of that for neutravidin and facilitates anti-biotin removal and renewal of the biotin binding sites if the surface is exposed to an excess of free biotin in solution.

**Figure 3.**
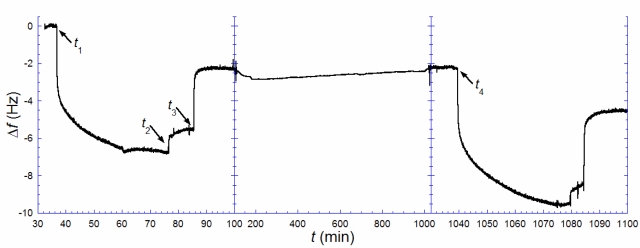
Changes in *f* upon addition of anti-biotin (*t*_1_ = 37 min) to an SLB from vesicles with biotin-lipids selectively incorporated in the outer leaflet. The exposure was followed by rinsing (*t*_2_ = 77 min) and addition of free biotin (*t*_3_ = 86 min) to promote removal of bound anti-biotin. Also shown is a second addition of anti-biotin (*t*_4_ = 1039 min) followed by rinsing (*t* = 1079 min) and addition of free biotin (*t* = 1085 min).

Figure 3 illustrates the results from such an experiment. A biotin-containing SLB, was exposed to anti-biotin (*t*_1_ = 37 min), followed by rinsing in pure buffer when the rate of binding had decreased significantly (*t*_2_ = 77 min). A solution of excess biotin (~150 μg/mL) was injected (*t*_3_ = 86 min) and kept in the cell until the rate of anti-biotin removal was zero. This step was followed by rinsing and storage in pure buffer up to time *t_4_* (= 1039 min). Note, however, that there is a remaining change in *f*, Δ*f*_1,irr_, of ~−2.2 Hz, after anti-biotin removal with excess biotin, signaling that a fraction of the antibiotin was irreversibly bound. This is likely attributed to a fraction of anti-biotin engaging both of their binding sites, which is known to increase the strength of binding by several orders of magnitude [23]. After several hours (*t*_4_ = 1039 min), an identical anti-biotin binding and removal procedure, as above, was performed. A total uptake of 9.6 Hz was registered, *i.e*., about 1.4 times larger than the uptake observed in the first anti-biotin exposure. Taking the irreversibly bound population of biotin-lipids into account, Equation (1) is transformed to:

(3) $$r_{a-b}=\frac{\Delta f_{1,\text{sat}}}{2(\Delta f_{2,\text{sat}}-\Delta f_{1,\text{irr}})+\Delta f_{1,\text{irr}}}$$

where Δ*f*_1,sat_ and Δ*f*_2,sat_ are the saturated amounts of anti-biotin bound after the 1^st^ (*t*_1_) and 2^nd^ (*t*_4_) additions respectively [24]. The measurement shown in Figure 3 yields *r*_a−b_ ≈ 0.4. This result again points towards a combination of scenarios (ii) (or (iv)) and (iii). Anti-biotin was also added a third time 27 h after the second rinse, but the total frequency shift did only slightly exceed that of the second addition, as expected if near-equilibrium had been reached at 16 h. The small additional uptake fits the shifted equilibrium from having a larger irreversibly bound anti-biotin fraction between the second and the third additions.

When analyzing these results, it is important to recall that SLB formation is more complicated than indicated in the schematics shown in Figure 1. As mentioned in the Introduction, the vesicle rupture is initiated by a combination of vesicle-surface and vesicle-vesicle interaction, while SLB growth is primarily controlled by the edges of the planar bilayer islands [16,17,25–27]. Thus, we cannot a priori exclude that there is one pathway dominating during the early stage of vesicle-vesicle initiated rupture and another during the edge-induced rupture phase. Since edge-induced rupture should dominate the process [12,17,28] the results should primarily be interpreted as pertaining to edge-induced rupture. It is clear that either pathway (ii) or (iv) must dominate during this process. However, since it is established that lipid desorption is negligible (less than 5% and concentrated to the final stage of the SLB formation process at this concentration) [12], scenario (iv) cannot be the dominating pathway during the primarily edge-induced rupture phase. This, in turn, leaves scenario (ii) as the most likely pathway for the major part of the SLB formation. The remaining pathway (iii) cannot be excluded from any part of the process on these grounds, but must be combined with pathway (ii) to obtain the observed low *r*-values (see further below). For example, if (iii) were the only rupture pathway, there would be no slow equilibration kinetics between monolayers *α* and *β*.

We stress that a value of *r* that does not exactly match any of the four proposed scenarios must not mean that the rupture process differs at different stages of the process. If only one pathway dominates throughout the whole process, there are still several factors and processes that can contribute to the observed 0 < *r* < 0.4, of which we will discuss the most important ones below.

First, we observed, in additional experiments, that if the time for formation of an SLB was reduced to several minutes by lowering the bulk vesicle concentration, *r* approached 0.5, *i.e*. the expected value if equilibration occurs between the upper and lower monolayers already during the SLB formation process. This suggests that either there is a strong component of scenario (iii), or that there is a transient diffusion of lipids between the upper and lower monolayers at the edge of SLB islands or at larger defects in the film already during the SLB formation process, which contributes to equilibration of the distribution of biotin-lipids. There is also a possible contribution from an increase in transient pore formation upon adsorption of liposomes, which could contribute to lipid mixing as recently demonstrated by Khan *et al.* [29]. When the rate of the SLB formation was increased (higher bulk concentration), the *r*-value could become as low as 0.2, which indicates a reduction of the transient equilibration process. Note that this transient equilibration will affect the observed *r*-value if scenario (ii) dominates, but would not change the experimental result if scenario (iii) dominates, since the latter has the same *r* as the equilibrium distribution. Diffusion of lipids around the edge of bilayer islands may however be far slower than expected from comparison with pore diffusion due to the remarkable stability observed for such edges by AFM indicating strong pinning [12,18,25–27,30].

Second, another factor of importance derives from the ethanol-based means for selective incorporation of lipids into monolayer *A* [31]. John *et al.* observed a flip-flop rate, which was a factor of ~300 higher when this process was used than typically quoted for the same lipid compositions [31–33]. This suggests that a small, but not negligible, fraction of biotin-lipids might reach the inner monolayer of the vesicles prior to surface exposure and rinsing. Even if scenario (ii) were dominating throughout the whole process, a measured value of *r* higher than zero would then be expected, while for scenario (iii) the obtained *r* would not be influenced.

Third, in contrast to the 2D schematics shown in Figure 1, the real 3D case implies that the membrane *cannot* unfold in a way that completely orients the outer monolayer towards the substrate. AFM images have shown that ruptured vesicles form rounded irregular shapes on SiO_2_ [12,18,30,34]. In essence, the transition from a domed deformed vesicle shape [14,15] to these irregular planar shapes must occur through a complex unfolding path, where some exchange of lipids between the two monolayers is likely. The edge-interface will be highly dynamic during rupture and the release of energy might increase the mixing of lipids from the two monolayers during unfolding.

Finally, there are two technical points that should be raised, which suggests that the obtained values of *r* are indeed overestimated. The first originates from that the neutravidin adsorption, and thus *r*_NA_, did not reach full equilibrium, as mentioned in the results section. The slow change in *r*_NA_ towards higher values is expected since the lipid distribution is continuously equilibrating over time with more rapid flip-flop to the upper monolayer for the membrane tested right after formation. Any small error in *r*_NA_ from this experimental complication would cause a higher than actual *r*_NA_ to be observed. The second technical point originates from conclusions drawn from combined SPR and QCM-D measurements of the binding of streptavidin (an analogue to neutravidin) to a biotin-SLB [35]. In brief, these data suggest that the amount of coupled water per adsorbed protein is significantly higher (more than a factor of 8) at low compared to high coverage. Thus, by using changes in *f* upon neutravidin (or anti-biotin) binding to get an estimate of the biotin-lipid concentration, an overestimation of biotin-lipids at low- compared with high coverage is expected. The ratios obtained from changes in *f* would thus, if this correction of unknown magnitude is included, yield lower values of *r*. The difference between *r*_NA_ and *r*_a−b_, with *r*_a−b_ > *r*_NA_ could also potentially be explained by the same effect. Anti-biotin will, due to its structure couple more water per molecule [36], which is likely to lead to an even higher variation as function of coverage.

Importantly, *all these factors* would, if pathway (ii) dominates*, lead to an increase in the observed r value*, well above zero. In contrast, they would have no influence on the *r* value for pathway (iii), which would still be 0.5. We thus find that *pathway (ii) is significantly strengthened* by these considerations, as the dominating rupture pathway during the major part of the SLB formation process.

In the following we discuss some additional data from the literature, in relation to the present results. Although pathway (ii) was observed by Puu and coworkers [18], they suggest (iii), which differs substantially in the end result to (ii), as the dominant pathway. However, a direct comparison between their work and the here presented is misleading since their observation of vesicle decomposition obtained with tapping-mode AFM is clearly do not correspond to those for small and large unilamellar phospholipid vesicles on oxide substrates by us and others. The time-scale in their experiment for single-vesicle rupture is orders of magnitude higher than observed for other systems under similar investigation [30,34,37]. Furthermore, the AFM-images show rupture taking place at a much lower local surface coverage of vesicles than found in other kinetic and AFM studies [12,34]. The most likely explanation for this striking difference is the inclusion of a high mol-% cholesterol, which is known to lead to radically different membrane mechanical properties [38,39] and consequently possibly to a different rupture pathway.

While scenarios (ii) and (iii) are physically related, they are both radically different from scenario (i), which is suggested from previous work on proteoliposomes. However, previous results on proteoliposomes are not necessarily in contradiction to the results on pure lipid systems presented here and previously [18,19]. The first study, performed by Contino *et al.* [10], while yielding a very clear-cut result pointing towards scenario (i), raises several questions in the light of more recent results on SLB formation. They used a protein with large hydrophilic domains uni-directionally incorporated in vesicles, but did not demonstrate that a complete SLB was actually formed. It is today known that for the highly negatively charged lipid mixture used in their work, the vesicle rupture and SLB formation is, under their buffer conditions, very weak on glass surfaces [13,40]. Actually, references on SLB formation for similar protocols given by Contino *et al.*, yield a lipid density on the surface more similar to that of a an adsorbed vesicle layer than that of an SLB [12,19,41]. If it is also taken into account that incorporated membrane proteins with large hydrophilic domains inhibit vesicle rupture [11] it is not unlikely that: (a) the rupture path might be different for these proteoliposomes compared to a pure lipid system and/or (b) that a majority-population of the adsorbed vesicles have not ruptured and the measured activity is that of surface bound vesicles. Thus, since the result for the protein activity demonstrated by Contino *et al.* is the same as expected for a vesicular layer, it is not unlikely that the response of vesicles instead of an SLB was measured [10,11]. In a later study, Salafsky *et al.* used a modified photosynthetic reaction center, with one flexible, hydrophilic part, which demonstrates high affinity to the surface [6]. These hydrophilic loops are situated on the opposite side of the transmembrane part of the protein in relation to the evaluated active site and has approximately the same thickness as the water-layer between the bilayer and the substrate [6]. All these factors aid in pinning the protein to the surface and orienting it with the active site towards the bulk during rupture, which would yield a protein directionality as if pathway (i) had been followed regardless of the actual process.

One suggestion for proteoliposome rupture, consistent with our conclusions, as well as with previous data, is that the membrane proteins do not necessarily follow the unfolding path of the lipid membrane. For example, upon adsorption of vesicles to the surface, the lipids, which have a higher diffusion coefficient than proteins, will diffuse in contact with and adsorb to the surface more rapidly than the proteins. If the proteins experience an attractive interaction with the surface, they will then accumulate at the edge of the adsorbed part of the membrane rather than accumulate unfavorably between the substrate and the membrane. Thus, when rupture/fusion of the vesicle occurs at a later stage, the proteins are in a position favorable for flipping even large hydrophilic domains without necessarily transferring them through the hydrophobic interior of the membrane. Then, if the active site is associated with these domains, it may end up facing the bulk even if monolayer *A* predominantly faces the substrate (becomes monolayer *β*) after SLB formation. The energy gain from such a positional reorientation of the proteins would not be as large for small proteins, which are accommodated in the membrane and would not cause pinning and bending of the bilayer, increasing the energy, when facing the substrate. We thus suggest that, while the outer lipid monolayer in the vesicle membrane will initially face the substrate after SLB formation, the orientation of incorporated proteins might not necessarily follow this path, but a path also influenced by their size and the strength of the surface interaction of their hydrophilic domains. Further experiments choosing a proper set of membrane proteins are needed to confirm or disprove this hypothesis, but an important result of this study is that the unfolding of the lipid membrane, despite being an important driving force for the reorientation of proteins on the substrate, is likely to often be decoupled from the protein redistribution trajectory.

## 3. Experimental Section

*Vesicle preparation*: Liposomes were produced by sonication of 1-palmitoyl-2-oleoyl-*sn*-glycero-3-phospocholine lipids (lyophilized POPC, Avanti Polar lipids) at a lipid concentration of 10 mg/mL in a bath sonicator (ULTRAsonik 28H, Ney) for 20 min, which produced large unilamellar vesicles approximately 100 nm in diameter determined by dynamic light scattering. To selectively modify the outer membrane for a population of vesicles, a protocol used by John *et al.* was modified [31]. A 1.5 mg/mL solution of POPC vesicles was incubated for ~1 h at 4 °C in buffer including 1 volume-% ethanol and 12.5–25 μg/mL dissolved 1,2-dipalmitoyl-sn-glycero-3-phosphoethanolamine-*N*-(cap biotinyl (biotin-PE, Avanti Polar Lipids). On this time-scale the biotin-lipids will insert in the outer monolayer, *A*, but will not have enough time to flip-flop to the inner monolayer, *B*, of the vesicles. The typical time-scale for transbilayer lipid exchange (including pore diffusion) in pure lipid vesicle membranes is many hours, even several days for phospholipids [31,32]. The vesicles, with biotin-lipids selectively incorporated in the outer monolayer, were used for SLB-formation immediately after the 1 h incubation.

*Chemicals*: standard buffer was 100 mM NaCl, 10 mM tris[hydroxymethyl]aminomethane (Tris, Sigma-Aldrich) made from Milli-Q water and set to pH 8.0 by addition of HCl. Other chemicals used were albumin (Sigma), neutravidin (lyophilized, Sigma), mouse IgG monoclonal 2F5 anti-biotin (Molecular Probes) and biotin (Sigma-Aldrich), used at final concentrations 15 μg/mL, 20 μg/mL, 6.6 μg/mL and ~150 μg/mL, respectively. The anti-biotin and biotin were dissolved in 137 mM NaCl PBS buffer, made from tablets (Sigma-Aldrich). The QCM sensor crystal surfaces sputter coated with SiO_2_ were cleaned by washing in 2 mM sodiumdodecylsulphate (SDS, Aldrich) solution and 2×10 min UV-ozone exposure.

*Quartz crystal microbalance with dissipation monitoring*: all measurements of the SLB formation, controls of non-specific binding and selective binding to the biotin-lipids in the supported bilayer were performed using the QCM-D technique [20]. Controls to make sure that the adsorption was due to specific binding of the neutravidin to biotin-lipids and not to surface defects were made by exposing pure POPC lipid bilayers prepared in the same way to neutravidin (20 mg/mL) and to albumin (15 mg/mL) before adsorption of neutravidin. Both controls showed that any non-specific protein binding is below the detection limit of the QCM-D (approximately 5 ng/cm^2^), as also previously observed for IgG and other water soluble proteins [3,42]. The QCM-D measures real-time kinetics through simultaneous measurements of two output signals from a piezoelectric quartz crystal oscillator sensor. The measured signals from the sensor crystal are the changes in resonant frequency, Δ*f*, and the change in energy dissipation, Δ*D* (or inverse Q-factor) in real time. Most measurements were performed at a single overtone, 15 MHz, of a crystal with a fundamental resonant frequency of 5 MHz. From a single-frequency measurement, the approximate adsorbed mass can be found from the Sauerbrey relation Δ*m* = −17.7 × Δ*f n* ng/cm^2^, where *n* is the overtone number, if the change in dissipation is low [43]. Importantly, the measured mass includes the mass of water, dynamically coupled to the oscillations of the adsorbed film. This water usually constitutes a large part of the measured Δ*f* and Δ*D* response and can vary with adsorbate coverage [35].

## 4. Conclusions

In summary, we have selectively modified the lipid composition in the outer monolayer of unilamellar liposome membranes and shown that for SLB formation on SiO_2_ surfaces the rupture process is dominated by pathway (ii) shown in Figure 1, which is the only scenario qualitatively compatible with the collected data. In this scenario, the bilayer membrane unfolds to expose the inner monolayer, *B*, of the vesicles predominantly to the bulk solution, as was shown to be the case to at minimum 70%. We have, however, also pointed out that there is most likely a transient mixing process between the inner and outer monolayers during the rupture and fusion process of vesicles. It was shown that the degree of this transient mixing process is influenced by the overall rate of SLB formation, by varying the bulk concentration of vesicles and becomes dominant if the SLB formation proceeds slowly. Even if this mixing is fast for lipids, the same is not necessarily true for incorporated proteins and thus the vesicle rupture pathway might still be very important for determining protein orientation in the SLB.

Comparing our observations and conclusions to previous results on incorporation of transmembrane proteins, a possible mechanism was suggested for how the orientation of proteins incorporated in vesicles is transferred to the SLB. This observation is of importance for the self assembly of, *e.g.*, membrane protein arrays predicted to be one of the major applications of such membranes. And an important conclusion from this comparison is that lipid membrane unfolding and membrane protein reorientation could be possible to control independently.
